# Programmed Intermittent Epidural Bolus vs Continuous Epidural Infusion for Labor Analgesia: A Comparative Study of Maternal Satisfaction and Breakthrough Pain

**DOI:** 10.7759/cureus.110560

**Published:** 2026-06-09

**Authors:** Maria Sgouraki, Pooja Sahu, Amiya Kumar Nayak, Heena Dixit, Jasmine Kalsi, Akriti Mahajan

**Affiliations:** 1 Department of Anesthesiology, Dr. Sulaiman Al Habib Hospital, Dubai, ARE; 2 Department of Obstetrics and Gynecology, Apollo Institute of Medical Sciences and Research, Hyderabad, IND; 3 Department of Anesthesiology, Pandit Raghunath Murmu Medical College and Hospital, Baripada, IND; 4 Department of Blood Cell, Commissionerate of Health and Family Welfare, Government of Telangana, Hyderabad, IND; 5 Department of General Surgery, Kalpana Chawla Government Medical College, Karnal, IND; 6 Department of Oral Medicine and Radiology, Desh Bhagat Dental College and Hospital, Mandi Gobindgarh, IND

**Keywords:** continuous epidural infusion, epidural analgesia, labor analgesia, maternal satisfaction, programmed intermittent epidural bolus

## Abstract

Introduction

Effective labor analgesia plays a crucial role in improving maternal comfort and the overall childbirth experience. The aim of the present study was to compare maternal satisfaction and breakthrough pain between programmed intermittent epidural bolus (PIEB) and continuous epidural infusion (CEI) techniques for labor analgesia.

Materials and methods

This cross-sectional study was conducted in the Department of Anesthesiology among 192 term pregnant women who received epidural labor analgesia. The participants were equally divided into PIEB (n = 96) and CEI (n = 96) groups according to the maintenance epidural regimen received during labor. Maternal satisfaction was assessed using a 0-10 numeric rating scale. Secondary outcomes included breakthrough pain, physician-administered rescue boluses, patient-controlled epidural analgesia (PCEA) utilization, analgesic consumption, adverse events, and willingness to repeat the same analgesic technique. Statistical analysis was performed with p < 0.05 considered statistically significant.

Results

Maternal satisfaction scores were significantly higher in the PIEB group than in the CEI group (8.6 ± 0.9 vs. 7.9 ± 1.2; p = 0.001). High satisfaction (score ≥8) was observed in 72 (75.0%) participants in the PIEB group compared with 56 (58.3%) in the CEI group. Breakthrough pain occurred less frequently in the PIEB group than in the CEI group (p = 0.011). The number of physician-administered top-up boluses and PCEA activation was significantly lower in the PIEB group. Total local anesthetic volume and fentanyl consumption were also significantly reduced in the PIEB group (p < 0.05). Adverse maternal events were low and comparable between the groups.

Conclusions

PIEB provided superior maternal satisfaction and improved analgesic efficacy with lower anesthetic consumption than CEI while maintaining a comparable safety profile. These findings support the use of PIEB as an effective maintenance strategy for epidural analgesia during labor.

## Introduction

Labor pain is one of the most intense forms of pain experienced by women during childbirth and can significantly influence maternal satisfaction, psychological well-being, and the overall childbirth experience. Effective labor analgesia not only provides pain relief but also contributes to improved maternal cooperation, reduced stress response, and enhanced obstetric outcomes [1]. Epidural analgesia remains the gold standard for labor pain management because of its superior analgesic efficacy, flexibility, and safety profile for both the mother and the fetus [2]. Over the years, various epidural maintenance techniques have been developed to optimize analgesic quality while minimizing drug consumption, motor blockade, and breakthrough pain [2].

Continuous epidural infusion (CEI) has traditionally been used as the standard maintenance technique for epidural analgesia. Although CEI provides stable analgesia, it may be associated with uneven spread of the local anesthetic, increased requirement for patient-controlled epidural analgesia (PCEA) boluses, higher local anesthetic consumption, and breakthrough pain episodes [3]. Programmed intermittent epidural bolus (PIEB) has emerged as an alternative technique that delivers local anesthetic in intermittent boluses at preset intervals, potentially improving epidural drug distribution and analgesic effectiveness [4]. Recent studies have suggested that PIEB may reduce breakthrough pain, decrease physician interventions, and improve maternal satisfaction compared with CEI [4,5].

Maternal satisfaction has become an important patient-centered outcome in obstetric anesthesia research, reflecting both the quality of analgesia and the overall childbirth experience. In addition to satisfaction, factors such as breakthrough pain occurrence, requirement for supplemental boluses, analgesic consumption, and willingness to repeat the same analgesic technique are clinically relevant parameters for evaluating epidural maintenance strategies [6]. However, evidence comparing PIEB and CEI in routine clinical practice remains limited in many settings.

Therefore, the aim of the present study was to compare maternal satisfaction and breakthrough pain between PIEB and CEI techniques for labor analgesia. The objectives of this study were to evaluate overall maternal satisfaction scores, occurrence of breakthrough pain, PCEA utilization, analgesic consumption, adverse events, and willingness to repeat the same epidural technique in future labor.

## Materials and methods

Study design, setting, and duration

This study was designed as a cross-sectional study that was conducted after the implementation of both analgesic maintenance techniques as routine clinical practice in the labor analgesia unit. No randomization or interventional allocation was performed, and participants were grouped according to the epidural maintenance technique routinely administered by the attending anesthesiologist or departmental protocol. This study was conducted in the Department of Anesthesiology at the Pandit Raghunath Murmu Medical College and Hospital, Baripada, India, from March 2023 to September 2023. The labor and delivery unit of the institution manages a high volume of obstetric cases annually and routinely provides epidural labor analgesia services for eligible participants. Data collection was performed in the postpartum ward within 24-48 hours of delivery.

Study population

The study population consisted of term pregnant women who requested epidural analgesia during labor and subsequently received either PIEB or CEI as a maintenance epidural regimen. A total of 192 participants were included in the study, with 96 in each group.

Eligibility criteria

Women were included in the study if they were 18 years or older, had a singleton pregnancy with cephalic presentation, completed at least 37 weeks of gestation, requested and received epidural analgesia during labor, and were willing to participate in the postpartum survey. Participants were excluded if they had contraindications to neuraxial analgesia, such as coagulopathy or local infection; underwent emergency cesarean delivery under epidural anesthesia; experienced epidural catheter replacement or conversion of technique during labor; had major maternal or fetal complications; or were unable to understand or complete the survey questionnaire because of language barriers or cognitive impairment.

Sample size calculation

Sample size estimation was performed a priori based on the primary outcome measure of maternal satisfaction, which was assessed using a 0-10 numeric rating scale. Obtaining a mean satisfaction score of 8.0 ± 1.5 in the CEI group and 8.7 ± 1.5 in the PIEB group from a previous study [7], with a minimal clinically important difference of 0.7, a statistical power of 80%, and a two-sided alpha error of 0.05, the minimum required sample size was calculated as 80 participants per group. Considering a possible 20% exclusion or incomplete survey rate, the final target sample size was increased to 96 participants in each group, resulting in 192 participants. Sample size calculations were performed using the G*Power software (version 3.9.7, Heinrich Heine University, Düsseldorf, Germany).

Epidural analgesia procedure

All epidural procedures were performed by experienced anesthesiologists, using standard aseptic precautions. An epidural catheter was inserted at either the L3-L4 or L4-L5 intervertebral space using the loss-of-resistance technique. After successful catheter placement, an initial loading dose of 10-15 mL of 0.125% bupivacaine combined with fentanyl 2 µg/mL was administered. Maternal hemodynamic parameters and fetal heart rate were continuously monitored throughout labor according to the institutional protocol.

Maintenance epidural regimens

Participants in the PIEB group received PIEBs consisting of 6 mL of 0.0625% bupivacaine with fentanyl 2 µg/mL administered every 60 min using an automated epidural pump. In addition, PCEA boluses of 5 mL with a lockout interval of 20 min were provided. Participants in the CEI group received CEI of the same analgesic solution at a rate of 8 mL/h along with identical PCEA settings consisting of 5 mL boluses and a 20-minute lockout interval. Both maintenance regimens were continued until delivery unless the clinical circumstances required modification or discontinuation of the epidural technique.

Data collection procedure

Within 24-48 hours after delivery, eligible participants were approached by a trained research nurse who was blinded to the group allocation during the survey administration. Data were collected using structured paper-based or electronic questionnaires (Appendix A). Demographic information, obstetric characteristics, epidural-related parameters, maternal satisfaction scores, breakthrough pain episodes, PCEA utilization, and willingness to repeat the same analgesic technique in future labor were recorded. Clinical data were obtained from the labor and anesthesia records.

Outcome measures

The primary outcome of this study was maternal satisfaction with the epidural analgesia. Satisfaction was assessed using a 0-10 numeric rating scale, where 0 indicated “extremely dissatisfied,” and 10 indicated “extremely satisfied” [8]. Participants were asked the question, “Overall, how satisfied were you with your labor epidural analgesia?” Secondary outcome measures included the occurrence of breakthrough pain, pain intensity during breakthrough episodes, requirement for physician-administered epidural top-up boluses, PCEA utilization, total local anesthetic consumption, total fentanyl dose administered, occurrence of motor block, adverse maternal events, and willingness to undergo the same epidural technique in future labor. Breakthrough pain was defined as the occurrence of any additional physician-administered epidural top-up during labor. Pain severity during breakthrough episodes was assessed using a 0-10 numeric rating scale [8].

Statistical analysis

All collected data were analyzed statistically (IBM SPSS Statistics for Windows, version 25.0 (released 2017; IBM Corp., Armonk, NY, USA)). Continuous variables were tested for normality using the Shapiro-Wilk test. Normally distributed variables are presented as mean ± SD and compared using the independent sample t-test. Non-normally distributed variables are expressed as medians with IQRs and compared using the Mann-Whitney U test. Categorical variables were expressed as frequencies and percentages and analyzed using Pearson’s chi-square test or Fisher’s exact test when the expected cell count was less than five. A two-sided p-value of less than 0.05 was considered statistically significant.

Ethical considerations

The study protocol was reviewed and approved by the institutional ethical committee (approval no. 6927/23) of the college prior to the commencement of the study. Written informed consent was obtained from all the participants prior to their inclusion in the study. This study was conducted in accordance with the ethical principles outlined in the Declaration of Helsinki. Confidentiality and anonymity of participant information were strictly maintained throughout the study. All collected data were anonymized and stored securely in password-protected electronic records that were accessible only to the research investigators.

## Results

A total of 192 participants were included in the study, with 96 (50.0%) in the PIEB group and 96 (50.0%) in the CEI group. The baseline demographic, obstetric, and epidural-related characteristics were comparable between the two groups (Table 1). The mean maternal age was 27.4 ± 3.8 years in the PIEB group and 27.9 ± 4.1 years in the CEI group (p = 0.342). Nulliparous women comprised 58 (60.4%) and 55 (57.3%) participants in the PIEB and CEI groups, respectively. Vaginal delivery occurred in 88 (91.7%) and 86 (89.6%) patients in the PIEB and CEI groups, respectively. No statistically significant differences were observed between the groups for any baseline variables (p > 0.05), indicating comparability of the study population.

**Table 1:** Baseline demographic, obstetric, and epidural characteristics of the study participants Data are presented as mean ± SD or as frequency, n (%). A p-value < 0.05 was considered statistically significant. Continuous variables were analyzed using the independent samples t-test, whereas categorical variables were analyzed using the Pearson chi-square test. CEI: continuous epidural infusion; df: degree of freedom; PIEB: programmed intermittent epidural bolus

Parameters	Variable	PIEB group (n = 96)	CEI group (n = 96)	Test values (df)	p-value
Maternal demographics	Age (years), mean ± SD	27.4 ± 3.8	27.9 ± 4.1	t = 0.876 (190)	0.342
	BMI (kg/m²), mean ± SD	24.8 ± 3.2	25.1 ± 3.4	t = 0.630 (190)	0.516
	Gestational age (weeks), mean ± SD	39.2 ± 1.1	39.0 ± 1.3	t = 1.151 (190)	0.228
Obstetric parameters	Nulliparous, n (%)	58 (60.4%)	55 (57.3%)	χ² = 0.194 (1)	0.659
	Cervical dilation at epidural (cm)	4.2 ± 1.3	4.5 ± 1.4	t = 1.538 (190)	0.118
	Duration of labor (hours), mean ± SD	8.6 ± 2.4	9.1 ± 2.7	t = 1.356 (190)	0.174
	Mode of delivery: Vaginal, n (%)	88 (91.7%)	86 (89.6%)	χ² = 0.245 (1)	0.612
Epidural technique parameters	Level of insertion: L3-L4, n (%)	54 (56.3%)	52 (54.2%)	χ² = 0.084 (1)	0.762
	Loading dose volume (mL), mean ± SD	12.4 ± 1.8	12.7 ± 1.9	t = 1.123 (190)	0.249

The maternal satisfaction scores were significantly higher in the PIEB group than in the CEI group (Table 2). The mean overall satisfaction score was 8.6 ± 0.9 in the PIEB group and 7.9 ± 1.2 in the CEI group (p = 0.001). High satisfaction scores (≥8) were reported by 72 (75.0%) participants in the PIEB group compared with 56 (58.3%) participants in the CEI group. Moderate satisfaction scores (5-7) were observed in 20 (20.8%) participants in the PIEB group and 31 (32.3%) in the CEI group, and poor satisfaction scores (≤4) were reported by 4 (4.2%) and 9 (9.4%) participants, respectively. These findings indicate superior maternal satisfaction with the PIEB technique.

**Table 2:** Comparison of maternal satisfaction scores between the PIEB and CEI groups Data are presented as mean ± SD, median (IQR), or frequency, n (%). * A p-value < 0.05 was considered statistically significant. Normally distributed continuous variables were analyzed using the independent samples t-test, whereas non-normally distributed variables were analyzed using the Mann-Whitney U test. Categorical variables were analyzed using the Pearson chi-square test. CEI: continuous epidural infusion; df: degree of freedom; NRS: numeric rating scale; PIEB: programmed intermittent epidural bolus

Outcome measure	PIEB (n = 96)	CEI (n = 96)	Test value (df)	p-value
Overall satisfaction score (0-10 NRS), mean ± SD	8.6 ± 0.9	7.9 ± 1.2	t = 4.57 (190)	0.001*
Median satisfaction score (IQR)	9 (8-9)	8 (7-9)	U = 3418 (190)	0.002*
Highly satisfied (score ≥8), n (%)	72 (75.0%)	56 (58.3%)	χ² = 6.00 (1)	0.016*
Moderately satisfied (score 5-7), n (%)	20 (20.8%)	31 (32.3%)	χ² = 3.23 (1)	0.073
Poorly satisfied (score ≤4), n (%)	4 (4.2%)	9 (9.4%)	χ² = 2.06 (1)	0.145

As presented in Table 3, breakthrough pain occurred significantly less frequently in the PIEB group than in the CEI group, affecting 22 (22.9%) and 38 (39.6%) patients, respectively (p = 0.011). Physician-administered top-up boluses were required by 18 (18.8%) participants in the PIEB group and 32 (33.3%) participants in the CEI group. The median PCEA activation was lower in the PIEB group, and fewer participants required at least one PCEA bolus. Furthermore, the willingness to repeat the same analgesic technique in future labor was significantly greater in the PIEB group than in the CEI group (p = 0.046).

**Table 3:** Comparison of breakthrough pain, PCEA utilization, and patient preference between the PIEB and CEI groups Data are presented as median (IQR) or frequency, n (%). * A p-value < 0.05 was considered statistically significant. Non-normally distributed variables were analyzed using the Mann-Whitney U test, whereas categorical variables were analyzed using the Pearson chi-square test. CEI: continuous epidural infusion; df: degree of freedom; NRS: numeric rating scale; PCEA: patient-controlled epidural analgesia; PIEB: programmed intermittent epidural bolus

Outcome measure		PIEB (n = 96)	CEI (n = 96)	Test value (df)	p-value
Breakthrough pain	Occurrence of breakthrough pain, n (%)	22 (22.9%)	38 (39.6%)	χ² = 6.21 (1)	0.011*
	Pain NRS at breakthrough, median (IQR)	5 (4-6)	6 (5-7)	z = 2.02 (190)	0.043*
	Physician top-up bolus required, n (%)	18 (18.8%)	32 (33.3%)	χ² = 5.30 (1)	0.023*
PCEA utilization	PCEA activations per patient, median (IQR)	1 (0-2)	2 (1-3)	z = 2.37 (190)	0.018*
	Patients using ≥1 PCEA bolus, n (%)	48 (50.0%)	63 (65.6%)	χ² = 4.81 (1)	0.028*
Patient preference	Willingness to repeat the same technique, n (%)	84 (87.5%)	73 (76.0%)	χ² = 4.23 (1)	0.046*

Table 4 shows that the PIEB group required significantly lower total local anesthetic volume (68.2 ± 11.4 mL vs. 75.6 ± 13.2 mL; p = 0.004) and lower total fentanyl dose (136.4 ± 22.8 µg vs. 151.2 ± 26.4 µg; p = 0.008) than the CEI group. Bilateral sensory block was achieved in 91 (94.8%) participants in the PIEB group and 88 (91.7%) participants in the CEI group, while motor block occurred in 9 (9.4%) and 14 (14.6%) participants, respectively. Adverse events such as hypotension, pruritus, and nausea/vomiting were low and comparable between the groups, with no statistically significant differences observed (p > 0.05).

**Table 4:** Comparison of analgesia quality parameters and adverse events between the PIEB and CEI groups Data are presented as mean ± SD or frequency, n (%). * A p-value < 0.05 was considered statistically significant. Continuous variables were analyzed using the independent samples t-test, whereas categorical variables were analyzed using the Pearson chi-square test. CEI: continuous epidural infusion; df: degree of freedom; PIEB: programmed intermittent epidural bolus; SBP: systolic blood pressure

Parameter		PIEB (n = 96)	CEI (n = 96)	Test value (df)	p-value
Analgesia quality	Total local anesthetic volume (mL), mean ± SD	68.2 ± 11.4	75.6 ± 13.2	t = 4.16 (190)	0.004*
	Total fentanyl dose (µg), mean ± SD	136.4 ± 22.8	151.2 ± 26.4	t = 4.16 (190)	0.008*
	Bilateral sensory block achieved, n (%)	91 (94.8%)	88 (91.7%)	χ² = 0.74 (1)	0.410
	Motor block (Bromage ≥1), n (%)	9 (9.4%)	14 (14.6%)	χ² = 1.24 (1)	0.261
Adverse events	Hypotension (SBP < 90 mmHg), n (%)	7 (7.3%)	6 (6.3%)	χ² = 0.08 (1)	0.779
	Pruritus, n (%)	11 (11.5%)	9 (9.4%)	χ² = 0.22 (1)	0.637
	Nausea/vomiting, n (%)	5 (5.2%)	7 (7.3%)	χ² = 0.36 (1)	0.544

## Discussion

Effective labor analgesia is an essential component of modern obstetric care, as it significantly influences maternal comfort, satisfaction, and overall childbirth experience. Epidural analgesia remains the gold standard for pain relief during labor because it provides superior analgesic efficacy while allowing the mother to remain awake and cooperative during delivery. The present study compared PIEB with CEI and demonstrated that PIEB was associated with improved maternal satisfaction, reduced breakthrough pain, lower PCEA requirements, and reduced local anesthetic consumption, without increasing adverse maternal events.

The higher maternal satisfaction observed with PIEB may be attributed to the more effective spread of the local anesthetic within the epidural space. Unlike CEI, which delivers medication at a slow continuous rate, PIEB administers intermittent boluses under higher pressure, resulting in a wider and more uniform distribution of anesthetic solutions [9]. This mechanism is likely to contribute to more consistent analgesia and fewer episodes of inadequate pain control. Maternal satisfaction is a multidimensional outcome influenced not only by pain relief but also by mobility, sense of control, and reduced need for additional physician interventions. Previous studies have similarly reported superior maternal satisfaction with PIEB techniques [10]. Wong et al. [11] demonstrated that PIEB improved analgesic quality and reduced breakthrough pain compared with conventional continuous infusion regimens. Likewise, Tzeng et al. [12] reported that PIEB resulted in better maternal comfort and lower anesthetic consumption during labor than CEI did.

Breakthrough pain remains one of the most clinically relevant limitations of labor epidural analgesia because it often necessitates additional physician-administered boluses and may negatively influence the childbirth experience. In the present study, breakthrough pain and physician rescue interventions were less frequent in the PIEB group. This finding is consistent with the observations of Capogna et al. [13], who suggested that intermittent bolus delivery maintains a more effective sensory blockade by promoting improved epidural spread. The lower pain scores during breakthrough episodes in the PIEB group further indicated that, even when analgesic failure occurred, the severity of pain was less pronounced [12]. The reduced incidence of breakthrough pain also decreases the anesthesiologist’s workload and may improve the workflow efficiency in busy obstetric units.

Another important finding of the present study was the lower requirement for PCEA activation and the reduced total local anesthetic and fentanyl consumption in the PIEB group. Lower anesthetic utilization is clinically beneficial because excessive local anesthetic administration may contribute to motor blockade, maternal immobility, urinary retention, and prolonged labor. PIEB appeared to optimize drug delivery efficiency by achieving adequate analgesia at smaller cumulative doses [14,15]. Similar reductions in anesthetic consumption were reported by Sia et al. [14] and McKenzie et al. [15], who demonstrated that PIEB regimens reduced hourly local anesthetic requirements while maintaining superior analgesic efficacy. Reduced opioid exposure may additionally minimize opioid-related adverse effects such as pruritus, nausea, and sedation.

Importantly, despite lower anesthetic consumption, the quality of analgesia in the PIEB group was not compromised. The rates of bilateral sensory block were comparable between the groups, while motor blockade remained low in both techniques. Preservation of motor function is particularly desirable during labor because it promotes maternal mobility and active participation during the second stage of labor [10]. The absence of significant differences in adverse maternal outcomes such as hypotension, nausea, vomiting, and pruritus further supports the safety profile of PIEB. Previous meta-analyses evaluating PIEB systems have similarly concluded that PIEB provides improved analgesia without increasing maternal or neonatal complications [10,12].

The findings of the present study have important clinical implications for obstetric anesthesia practice. Implementation of PIEB as a routine maintenance strategy for labor epidural analgesia may improve patient-centered outcomes while simultaneously reducing physician intervention and anesthetic drug consumption. In high-volume obstetric centers, reduced breakthrough pain and fewer rescue boluses may help optimize staff workload and improve the overall efficiency of labor analgesia services. Furthermore, enhanced maternal satisfaction and willingness to repeat the same analgesic technique in future pregnancies indicate improved patient acceptance of epidural analgesia.

However, certain additional limitations of the present study should be considered while interpreting the findings. First, the non-randomized observational design may have introduced selection bias because allocation to the PIEB or CEI group was determined according to anesthesiologist preference and departmental practice protocols rather than random assignment. Although all epidural procedures were performed by experienced anesthesiologists, individual operator expertise and procedural variations were not separately analyzed, which may have influenced analgesic outcomes. Variability in epidural catheter insertion level, with catheters inserted at either the L3-L4 or L4-L5 intervertebral space, could also have affected anesthetic spread and analgesic efficacy. In addition, obstetric variables such as the degree of perineal tears and detailed labor characteristics were not comparatively evaluated and may have influenced postpartum maternal satisfaction scores.

Maternal satisfaction was assessed within 24-48 hours postpartum using a subjective self-reported numeric rating scale and may therefore have been affected by recall bias, emotional state, and overall childbirth experience. Breakthrough pain was defined according to physician-administered rescue epidural boluses, and differences in clinician judgment regarding rescue bolus administration may have introduced inter-observer variability. Furthermore, although baseline characteristics between groups were comparable, no multivariable adjustment analysis was performed to control for potential residual confounding factors. The study also did not document the use of additional non-epidural analgesic interventions, such as intravenous analgesics, transcutaneous electrical nerve stimulation, or other supportive pain-relief modalities that could potentially influence maternal pain perception and satisfaction outcomes. Finally, the single-center design may limit the external generalizability of the findings to other obstetric populations and institutional settings. Further multicenter randomized controlled trials with standardized analgesic protocols, assessment of neonatal outcomes, and long-term maternal satisfaction measures are recommended to further validate the superiority and broader applicability of PIEB over CEI techniques.

## Conclusions

PIEB demonstrated superior clinical performance compared to CEI for the maintenance of labor analgesia. PIEB was associated with higher maternal satisfaction, reduced breakthrough pain, lower PCEA utilization, and decreased local anesthetic and fentanyl consumption while maintaining a comparable safety profile. The technique also reduced the requirement for physician-administered rescue boluses, suggesting improved analgesic efficiency and workflow optimization in obstetric anesthesia practice. Despite similar rates of adverse maternal events, participants receiving PIEB expressed a greater willingness to repeat the same analgesic technique in future labor, supporting its potential role as a preferred maintenance strategy for labor epidural analgesia.

## Appendices

Appendix A: Postpartum Maternal Satisfaction and Labor Analgesia Questionnaire

Study title: Programmed Intermittent Epidural Bolus versus Continuous Epidural Infusion for Labor Analgesia: A Comparative Study of Maternal Satisfaction and Breakthrough Pain

Instructions: This questionnaire was administered within 24-48 hours after delivery. Please answer all questions based on your experience with epidural labor analgesia during your recent labor and delivery.

Section A. Participant information

1. Age (years): ________

2. Gravidity: ________

3. Parity: ________

4. Gestational age at delivery (weeks): ________

5. Mode of delivery:□ Vaginal delivery □ Assisted vaginal delivery □ Cesarean delivery

Section B. Maternal satisfaction with epidural analgesia

6. Overall, how satisfied were you with your labor epidural analgesia?

0   1   2   3   4   5   6   7   8   9   10

Extremely dissatisfied ----------------- Extremely satisfied

7. How would you rate the effectiveness of pain relief provided by the epidural?

□ Very poor □ Poor □ Fair □ Good □ Very good □ Excellent

8. Did the epidural help you remain comfortable during labor?

□ Strongly disagree o Disagree □ Neutral □ Agree □ Strongly agree

Section C. Breakthrough pain assessment

9. Did you experience any episode of pain that required additional intervention after epidural analgesia was established?

□ Yes □ No

10. If yes, please rate the worst breakthrough pain you experienced.

0   1   2   3   4   5   6   7   8   9   10

No pain ----------------- Worst imaginable pain

Section D. Patient-controlled epidural analgesia (PCEA)

11. Did you use the PCEA button during labor? □ Yes □ No

12. If yes, approximately how many times did you activate the PCEA button?

□ 1-2 times □ 3-4 times □ More than 4 times □ Do not remember

Section E. Adverse effects

13. Did you experience any of the following after receiving epidural analgesia?

□ Nausea         Yes/No

□ Vomiting      Yes/No

□ Itching (pruritus)       Yes/No

□ Dizziness      Yes/No

□ Weakness in legs      Yes/No

□ Any other symptom  Yes/No

If yes, please specify: ________

Section F. Future preference

14. If you have another childbirth in the future, would you choose the same epidural analgesia technique again?

□ Definitely yes □ Probably yes □ Not sure □ Probably no □ Definitely no

15. Would you recommend epidural labor analgesia to other pregnant women?

□ Yes □ No □ Not sure

16. Additional comments: _____________________________________

Scoring used in the study

Overall maternal satisfaction was assessed using a 0-10 Numeric Rating Scale (NRS). High satisfaction: score 2:8; Moderate satisfaction: score 5-7; Poor satisfaction: score ≤4. Breakthrough pain severity was assessed using a 0-10 NRS pain scale.
